# Prevalence and Diagnosis of Diabetic Cardiovascular Autonomic Neuropathy in Beijing, China: A Retrospective Multicenter Clinical Study

**DOI:** 10.3389/fnins.2019.01144

**Published:** 2019-10-25

**Authors:** Qi Pan, Quanmin Li, Wei Deng, Dong Zhao, Lin Qi, Wei Huang, Li Ma, Hongmei Li, Yufeng Li, Xiaofeng Lyu, Aihong Wang, Hebin Yao, Lixin Guo, Xiaoyan Xing

**Affiliations:** ^1^Department of Endocrinology, National Center of Gerontology, Beijing Hospital, Beijing, China; ^2^The PLA Rocket Force Characteristic Medical Center, Beijing, China; ^3^Department of Endocrinology, Beijing Jishuitan Hospital, Beijing, China; ^4^Center for Endocrine Metabolism and Immune Diseases, Luhe Hospital, Capital Medical University, Beijing, China; ^5^Department of Endocrinology, Beijing Yanhua Hospital, Beijing, China; ^6^Department of Endocrinology, Beijing Haidian Hospital, Beijing, China; ^7^South Section, Department of Endocrinology, Guang’anmen Hospital, China Academy of Chinese Medical Sciences, Beijing, China; ^8^Department of Endocrinology, Emergency General Hospital, Beijing, China; ^9^Department of Endocrinology, Beijing Pinggu Hospital, Beijing, China; ^10^Department of Endocrinology, Seventh Medical Center of PLA General Hospital, Beijing, China; ^11^PLA Strategic Support Force Characteristic Medical Center, Beijing, China; ^12^Department of Endocrinology, Sixth Medical Center of PLA General Hospital, Beijing, China; ^13^Department of Endocrinology, China-Japan Friendship Hospital, Beijing, China

**Keywords:** cardiovascular autonomic neuropathy, diabetes mellitus, risk factors, diagnostic method, a Multicenter Clinical Study

## Abstract

Cardiovascular autonomic neuropathy (CAN) is a debilitating condition occurring among diabetic patients especially those with long duration of disease. Whereas incidences and treatment of CAN has been well described for Western populations, fewer studies have been conducted among the Chinese. This study, therefore, aimed to assess the prevalence of CAN among sampled Chinese diabetic patients. Accordingly, 2,048 participants with a history of type 1 diabetes mellitus (T1DM, 73) and type 2 diabetes mellitus (T2DM, 1975) were randomly sampled from 13 hospitals. Patients’ biodata were recorded, and autonomic nervous system function tests performed to aid in the preliminary diagnosis of CAN. The final CAN diagnosis was based on the Ewing’s test in which heart rate variation (HRV) values were evaluated through deep-breathing (DB), lying-to-standing (LS), and Valsalva (V) tests. Systolic blood pressure (SBP) variation values were also evaluated through LS. In the T1DM group, 61.6% patients were diagnosed with CAN and no differences were observed in the baseline and clinical data between this group and those without CAN (*P* > 0.05). In the T2DM group, 62.6% patients were diagnosed with CAN and statistically significant differences were found between the CAN and non- CAN group with regards to age, duration of diabetes, metformin treatment, retinopathy, and hypertension history (*P* < 0.05). The most common manifestations of CAN included weakness (28.6%), dizziness (23.4%), frequent urination (19.6%), upper body sweating (18.3%), and nocturia (15.9%). Additionally, duration of disease and age were independent risk factors for CAN in T1DM and T2DM, respectively. On diagnosis, a combination of the V test + LS test provided the highest sensitivity of detecting CAN among T1DM group (sensitivity = 97.6%, AUC = 0.887) while for T2DM category, DB test had the highest sensitivity (83.6%), and maximal AUC (0.856) was found with V test + DB test. The overall prevalence of diabetes with CAN in the study was up to 63%.

## Introduction

Cardiovascular autonomic neuropathy (CAN) is one of the most serious diabetic complications but is often unrecognized by patients and clinicians (Vinik and Ziegler, 2007; Chen et al., 2015). Varying incidence and prevalence of CAN are reported in different studies among diabetic patients with the rates ranging from as low as 1.6% in patients with well-controlled diabetes to as high as 90% in those awaiting a pancreas transplant (Vinik et al., 2013).

Dysfunctions of the autonomic nervous system among CAN patients result in impaired cardiovascular regulation (Cha et al., 2016). Consequently, diabetic patients with undiagnosed CAN have increased cardiovascular risks that can be suddenly fatal (Hazari et al., 2012).

The development of CAN is reported to be associated with poor glycemic control, duration of diabetes, old age, female gender, and lifestyle factors such as smoking (Maser and Lenhard, 2005; Pop-Busui, 2012). However, there is wide heterogeneity in the causes and progression of CAN among diabetics (Pop-Busui, 2012).

Assessing patients for the presence of CAN is generally based on stimulating autonomic physiological functions and observing end-organ reactions. The tests include evaluating heart rate variations (HRV) and blood pressure changes (Khoharo and Halepoto, 2012). Some of the techniques employed in assessing HRV are deep-breathing (DB), lying-to-standing (LS), and Valsalva (V) maneuvers; these reflect parasympathetic function. On the other hand, measuring blood pressure changes during orthostatis, Valsalva (V) maneuvers and sustained isometric muscular strain aid in evaluating sympathetic function (Ewing et al., 1985). There is no specific algorithm for detecting CAN but, for proper diagnosis of the condition, it is recommended that more than one test is conducted to improve the sensitivity and reliability of the detection (Philips et al., 2011; Pop-Busui et al., 2017; Didangelos et al., 2018). The present study was undertaken with the aim of evaluating the prevalence of CAN among diabetic patients in Beijing, China. A further purpose of the study was to evaluate the specificity and sensitivity of the various functional tests in the assessment of CAN.

## Materials and Methods

### Subjects

Data of diabetic patients undergoing cardiac autonomic neuropathy assessment was collected retrospectively from 13 hospitals in Beijing. From each hospital 160 cases were randomly sampled over a period of 12 weeks. A randomly selected day every week was used as a day of investigation. The inclusion criteria for the study were as follows: (1) Meeting T1DM or T2DM diagnosis standards established by the World Health Organization [WHO] (1999). (2) Patient maintaining appropriate attention throughout the study. (3) Ability to understand study instructions and cooperate in completing the assessment. The exclusion criteria were: (1) Existence of other causes of neuropathy such as cervical lumbar lesions, cerebral infarction, and Guillain-Barre syndrome. (2) Patients who had serious arteriovenous vascular lesions. (3) Patients with neurotoxic effects caused by drugs in the setting of renal insufficiency. (4) Patients who were taking beta-blocker drugs. (5) Pregnant or breast-feeding women. (6) Patients with mental illness. (7) Patients who were reluctant to cooperate with study instructions. The study was approved by the institutional review boards at each study site and informed consent was obtained from all participants.

### CAN Evaluation

Sympathetic and parasympathetic function tests were performed to provide preliminary diagnosis of CAN. Standardized CAN evaluation was conducted by four non-invasive cardiovascular autonomic function tests as previously described (May and Arildsen, 2000; Pafili et al., 2015). In our study, a diagnosis of CAN was confirmed based on abnormal results in any two of the four tests described hereafter.

#### Deep-Breathing (DB) Test

Following 20-min acclimatization, the patient was asked to sit calmly and take 6 breaths, deeply and discreetly, over a period of 1 min. An electrocardiogram (ECG) reading was taken throughout this period. Additionally, the maximum and minimum beat-to-beat (R-R) intervals were recorded and beats per minute were derived from this value. The induced heart rate change was determined by calculating the mean of the difference between minimum and maximum heart rates during 6 rounds of the DB test. A difference of ≥15 beats/min was considered normal; 11–14 beats/min was taken to be borderline while a value of ≤10 beats/min was considered abnormal.

#### Lying-to-Standing (LS) Test

The patient was requested to lie in a supine position and an ECG recording was taken. Thereafter, the patient was asked to stand up. The maximum R-R interval close to the 15th beat and the minimum R-R interval close to the 30th beat was obtained. From these, the R-R ratio at the 30th and 15th beats (30/15 ratio) was calculated. A ratio ≥1.04 was considered normal; 1.01–1.03 was identified as borderline, and ≤1.00 was considered abnormal.

#### Valsalva (V) Tests

The patient was asked to sit in a relaxed state and blow hard to make the mercury sphygmomanometer rise to 40 mmHg for 15s before releasing pressure while an ECG reading was being taken. Differences in the heart rate evoked by the Valsalva maneuver were measured as the ratio of the highest tachycardia during the maneuver to the lowest bradycardia after the maneuver. This Valsalva ratio was presented as the ratio of maximum R-R interval after the Valsalva maneuver to minimum R-R interval during the Valsalva maneuver. A ratio of ≥1.21 was considered normal; 1.11–1.20 was taken to be borderline, and ≤1.10 was considered abnormal.

#### Change in SBP in Response to Lying-to-Standing Positions

The patient was asked to lie calmly in a supine position and then to stand up while blood pressure was monitored. A drop in SBP by ≤10 mmHg in response to standing was considered normal, whereas a fall in SBP of ≥30 mmHg was taken to be abnormal.

### Statistical Analysis

Continuous data with a normal distribution were displayed as means and standard deviations and compared using the independent *t*-test. Continuous data with a skewed distribution were displayed as medians and interquartile ranges and compared using the Wilcoxon rank-sum test. Categorical data were presented as frequencies or percentages and compared using the chi-square test. Clinical characteristics were compared between those with and without CAN. Multivariate logistic regression analysis was performed to estimate the risk factors for developing CAN. The risk factors that differed significantly between the CAN and the non-CAN patients were included in the model. In addition, age and gender were also entered into the model as adjustment variables. The receiver operating characteristic curve was then used to evaluate the performance of the 4 tests both in isolation and their respective combinations in the diagnosis of CAN to determine the optimal diagnostic method (DeLong et al., 1988). A value of *P* < 0.05 was considered statistically significant. The SAS software version 9.2 was utilized for all analyses.

## Results

The study recruited 2,048 subjects that were categorized into T1DM (*n* = 73) and T2DM (*n* = 1975) groups. The general characteristics of the two groups with respect to their CAN status are presented in Table 1. The prevalence of CAN in T1DM and T2DM was 61.6% and 62.6%, respectively. In contrast to T1DM patients, CAN patients suffering from T2DM had significant differences (*P* < 0.01), in comparison to patients without CAN, in terms of age, education, childbearing history and medical payment.

**TABLE 1:** General characteristics of T1DM and T2DM patients by CAN status.

	**T1DM**				**T2DM**			
	**CAN (*n* = 45)**	**Non-CAN (*n* = 28)**	**χ^2^/*t***	***P***	**CAN (*n* = 1236)**	**Non-CAN (*n* = 739)**	**χ^2^/*t***	***P***
Gender								
Male, *n* (%)	24 (53.33)	15 (53.57)	<0.001	0.984	604 (48.87)	389 (52.64)	2.631	0.105
Female, *n* (%)	21 (46.67)	13 (46.43)			632 (51.13)	350 (47.36)		
Age, year	52.96 ± 13.30	54.57 ± 12.98	−0.509	0.612	60.20 ± 10.63	57.14 ± 10.97	−6.116	<0.001^∗∗∗^
<60 years, *n* (%)	25 (55.56)	13 (46.43)	0.576	0.448	548 (44.34)	408 (55.21)	21.893	<0.001^∗∗∗^
≥60 years, *n* (%)	20 (44.44)	15 (53.57)			688 (55.66)	331 (44.79)		
BMI								
<24, *n* (%)	19 (42.22)	10 (35.71)	1.459	0.482	413 (33.41)	249 (33.69)	0.783	0.676
24–27.99, *n* (%)	18 (40.00)	15 (53.57)			557 (45.06)	343 (46.41)		
≥28, *n* (%)	8 (17.78)	3 (10.71)			266 (21.52)	147 (19.89)		
Education, *n* (%)								
Middle school or below	20 (44.44)	8 (28.57)	2.401	0.301	545 (44.09)	243 (32.88)	24.724	<0.001^∗∗∗^
High school	14 (31.11)	9 (32.14)			344 (27.83)	237 (32.07)		
Colleges and universities	11 (24.44)	11 (39.29)			347 (28.07)	259 (35.05)		
Child-bearing history, *n* (%)								
0	5 (11.11)	3 (10.71)	0.059	0.971	71 (5.74)	50 (6.77)	7.866	0.020^∗^
1	22 (48.89)	13 (46.43)			713 (57.69)	464 (62.79)		
≥2	18 (40.00)	12 (42.86)			452 (36.57)	225 (30.45)		

*Data are mean ± SD or n (%), *P* value is calculated by the Wilcoxon rank sum test or the χ2 test comparing CAN vs. non-CAN. ^∗^*P* < 0.05; ^∗∗∗^*P* < 0.001.*

As shown in Tables 2, 3, there were observable differences in patient characteristics and medication history between the cohorts. Parameters correlating with CAN among T1DM include longer duration disease (*z* = 2.131, *P* = 0.033) and longer metformin medication use (*z* = 3.059, *P* = 0.002). On the other hand, among T2DM patients, correlation with CAN was seen with longer duration of disease (*z* = −4.204, *P* < 0.001), higher hypoglycemic level (*z* = −2.200, *P* = 0.028), larger metformin dosage (*z* = −2.858, *P* = 0.004) and longer metformin medication time (*z* = −4.364, *P* < 0.001).

**TABLE 2:** Patient history and complications of T1DM and T2DM groups by CAN status.

	**T1DM**				**T2DM**			
	**CAN (45)**	**Non-CAN (28)**	**χ^2^/z**	***P***	**CAN (1236)**	**Non-CAN (739)**	**χ^2^/z**	***P***
Course of disease, year, median (IQR)	12 (3–17)	5 (3–11)	2.131	0.033^∗^	10 (4–15)	7.5 (3–12)	–4.204	< 0.001^***^
<5 years, *n* (%)	13 (28.89)	10 (35.71)	6.952	0.031^∗^	351 (28.40)	247 (33.42)	15.277	< 0.001^***^
5–10 years, *n* (%)	5 (11.11)	9 (32.14)			241 (19.50)	174 (23.55)		
≥10 years, *n* (%)	27 (60.00)	9 (32.14)			644 (52.10)	318 (43.03)		
HbA1c, median (IQR)	8 (7–9.4)	7.3 (6.5–8.3)	1.612	0.107	7.6 (6.7–8.99)	7.3 (6.5–8.6)	–2.200	0.028^∗^
Hypoglycemia								
Yes, *n* (%)	16 (35.56)	7 (25.00)	0.891	0.345	188 (25.44)	328 (26.54)	0.289	0.591
No, *n* (%)	29 (64.44)	21 (75.00)			551 (74.56)	908 (73.46)		
Metformin								
Yes, *n* (%)	25 (55.56)	15 (53.57)	0.027	0.868	434 (58.73)	755 (61.08)	1.072	0.301
No, *n* (%)	20 (44.44)	13 (46.43)			305 (41.27)	481 (38.92)		
Metformin dosage in g, median (IQR)	1.5 (1.5–1.5)	1.5 (0.125–1.5)	1.681	0.093	1.5 (1–2)	1.5 (0.5–1.5)	–2.858	0.004^∗∗^
Duration of medication, month, median (IQR)	96 (30–161)	16 (1–66)	3.059	0.002^∗∗^	48 (8–120)	24 (1–96)	–4.364	< 0.001^***^
Kidney disease								
Yes, *n* (%)	9 (20.00)	0 (0.00)	6.388	0.011^∗^	107 (8.66)	48 (6.50)	2.988	0.084
No, *n* (%)	36 (80.00)	28 (100.00)			1129 (91.34)	691 (93.50)		
Retinopathy								
Yes, *n* (%)	11 (24.44)	0 (0.00)	8.059	0.005^∗∗^	274 (22.20)	137 (18.56)	3.710	0.054
No, *n* (%)	34 (75.56)	28 (100.00)			960 (77.80)	601 (81.44)		
Coronary heart disease								
Yes, *n* (%)	2 (4.44)	0 (0.00)	1.280	0.258	211 (17.14)	90 (12.23)	8.576	0.003^∗∗^
No, *n* (%)	43 (95.56)	28 (100.00)			1020 (82.86)	646 (87.77)		
Cerebral infarction								
Yes, *n* (%)	1 (2.22)	0 (0.00)	0.631	0.427	135 (10.99)	69 (9.35)	1.340	0.247
No, *n* (%)	44 (97.78)	28 (100.00)			1093 (89.01)	669 (90.65)		
Diabetic neuropathy								
Yes, *n* (%)	8 (17.78)	5 (17.86)	<0.001	0.993	153 (12.40)	93 (12.58)	0.015	0.904
No, *n* (%)	37 (82.22)	23 (82.14)			1081 (87.6)	646 (87.42)		

*Data are medians and interquartile ranges or n (%), *P* value is calculated by the Wilcoxon rank sum test or the χ2 test comparing CAN vs. non-CAN. ^∗^*P* < 0.05; ^∗∗^*P* < 0.01; ^∗∗∗^*P* < 0.001. IQR, interquartile range.*

**TABLE 3:** Symptoms of T1DM and T2DM groups by CAN status.

	**T1DM**				**T2DM**			
	**CAN (45)**	**Non-CAN (28)**	**χ^2^**	***P***	**CAN (1236)**	**Non-CAN (739)**	**χ^2^**	***P***
**Tumble**								
Yes, *n* (%)	0 (0.00)	0 (0.00)	–	–	80 (6.53)	38 (5.15)	1.556	0.212
No, *n* (%)	45 (100.00)	28 (100.00)			1145 (93.47)	700 (94.85)		
**Dizziness, instability**								
Yes, *n* (%)	13 (100.00)	3 (60.00)	5.850	0.016^∗^	317 (66.74)	157 (55.48)	9.600	0.002^∗∗^
No, *n* (%)	0 (0.00)	2 (40.00)			158 (33.26)	126 (44.52)		
**Dyspnea**								
Yes, *n* (%)	0 (0.00)	0 (0.00)	–	–	44 (21.67)	22 (14.86)	2.600	0.107
No, *n* (%)	0 (0.00)	2 (100.00)			159 (78.33)	126 (85.14)		
**Weak**								
Yes, *n* (%)	13 (100.00)	9 (81.82)	2.579	0.108	355 (69.07)	192 (60.38)	6.586	0.010^∗^
No, *n* (%)	0 (0.00)	2 (18.18)			159 (30.93)	126 (39.62)		
**Coma**								
Yes, *n* (%)	0 (0.00)	0 (0.00)	–	–	5 (3.05)	4 (3.08)	<0.001	0.989
No, *n* (%)	0 (0.00)	2 (100.00)			159 (96.95)	126 (96.92)		
**Postprandial fullness**								
Yes, *n* (%)	6 (85.71)	1 (33.33)	2.744	0.098	192 (44.92)	107 (36.9)	4.625	0.032^∗^
No, *n* (%)	1 (14.29)	2 (66.67)			235 (55.04)	183 (63.10)		
**Nausea**								
Yes, *n* (%)	4 (80.00)	1 (33.33)	1.742	0.187	70 (22.95)	40 (17.94)	1.963	0.161
No, *n* (%)	1 (20.00)	2 (66.67)			235 (77.05)	183 (82.06)		
**Emesis**								
Yes, *n* (%)	1 (50.00)	0 (0.00)	1.333	0.248	22 (8.56)	16 (8.04)	0.040	0.842
No, *n* (%)	1 (50.00)	2 (100.00)			235 (91.44)	183 (91.96)		
**Epigastric pain**								
Yes, *n* (%)	0 (0.00)	2 (50.00)	0.833	0.361	23 (8.91)	17 (8.50)	0.024	0.876
No, *n* (%)	1 (100.00)	2 (50.00)			235 (91.09)	183 (91.50)		
**Dysuria**								
Yes, *n* (%)	0 (0.00)	0 (0.00)	–	–	73 (28.63)	43 (21.94)	2.595	0.107
No, *n* (%)	1 (100.00)	2 (100.00)			182 (71.37)	153 (78.06)		
**Frequent urination**								
Yes, *n* (%)	8 (88.89)	6 (75.00)	0.562	0.453	253 (58.16)	113 (42.48)	16.265	<0.001^∗∗∗^
No, *n* (%)	1 (11.11)	2 (25.00)			182 (41.84)	153 (57.52)		
**Nocturia**								
Yes, *n* (%)	14 (93.33)	10 (83.33)	0.675	0.411	209 (53.45)	96 (38.55)	13.536	<0.001^∗∗∗^
No, *n* (%)	1 (6.67)	2 (16.67)			182 (46.55)	153 (61.45)		
**Urgent urination**								
Yes, *n* (%)	3 (75.00)	4 (66.67)	0.079	0.778	140 (43.61)	59 (27.83)	13.595	<0.001^∗∗∗^
No, *n* (%)	1 (25.00)	2 (33.33)			181 (56.39)	153 (72.17)		
**Sexual function**								
Satisfaction, *n* (%)	2 (66.67)	2 (66.67)	–	–	62 (24.41)	42 (22.34)	0.257	0.612
Dissatisfaction, *n* (%)	1 (33.33)	1 (33.33)			192 (75.59)	146 (77.66)		
**Upper body sweating**								
Yes, *n* (%)	13 (100.00)	4 (80.00)	2.753	0.097	250 (56.56)	127 (46.52)	6.826	0.009^∗∗^
No, *n* (%)	0 (0.00)	1 (20.00)			192 (43.44)	146 (53.48)		
**Head and neck sweating**								
Yes, *n* (%)	2 (100.00)	3 (75.00)	0.600	0.439	98 (33.79)	52 (26.26)	3.134	0.077
No, *n* (%)	0 (0.00)	1 (25.00)			192 (66.21)	146 (73.74)		

*Data are *n* (%), *P* value is calculated using the χ^2^ test comparing CAN vs. non-CAN. ^∗^*P* < 0.05; ^∗∗^*P* < 0.01; ^∗∗∗^*P* < 0.001.*

With reference to disease symptoms and complications, kidney disease (χ^2^ = 6.388, *P* = 0.011), retinopathy (χ^2^ = 8.059, *P* = 0.005), dizziness and instability (χ^2^ = 5.85, *P* = 0.016) were the most correlated with CAN among T1DM patients. In comparison, among T2DM patients, CAN was associated with coronary heart disease (χ^2^ = 8.576, *P* = 0.003), peripheral vascular disease (χ^2^ = 5.759, *P* = 0.016) and dizziness, instability (χ^2^ = 9.600, *P* = 0.002). Other symptoms were weakness (χ^2^ = 6.586, *P* = 0.010), postprandial fullness (χ^2^ = 4.625, *P* = 0.032), frequent urination (χ^2=^16.265, *P* < 0.001), nocturia (χ^2^ = 13.536, *P* ≤ 0.001), urgent urination (χ^2^ = 13.595, *P* < 0.001) and upper body sweating (χ^2^ = 6.826, *P* = 0.009).

Considering both T1DM and T2DM groups, the most frequent symptoms of CAN were weakness (28.6%), dizziness (23.4%), frequent urination (19.6%), upper body sweating (18.4%), and nocturia (15.9%). The specificity of all the above symptoms was poor since they may occur in other diseases, thus necessitating further screening.

In view of the past medical history of diabetic patients, we found that the proportion of CAN patients with hypertension was higher than that in non-CAN patients in T2DM (χ^2^ = 11.886, *P* = 0.001). Interestingly, the serum levels of triglycerides (TG) of CAN patients were lower than that in non-CAN patients (χ^2^ = 2.273, *P* = 0.023).

To evaluate the relative contribution of individual risk factors for CAN in both patient cohorts, we performed regression analysis. In T1DM, duration of disease was an independent risk factor for developing CAN (OR = 1.122, 95% CI 1.026–1.228, *P* = 0.011) (Table 4) whereas in T2DM, age was an independent risk factor (OR = 1.040, 95% CI 1.008–1.072, *P* = 0.012) (Table 5).

**TABLE 4:** Risk factors for CAN in TIDM patients.

	**OR**	**95% CI lower**	**95% CI upper**	***P***
Gender	1.610	0.536	4.837	0.396
Age	0.966	0.924	1.009	0.123
Duration of disease (year)	1.122	1.026	1.228	0.011
Hba1c	1.151	0.845	1.566	0.372

*A value of *P* < 0.05 was considered to be statistically significant.*

**TABLE 5:** Risk factors for CAN in T2DM patients.

	**OR**	**95% CI lower**	**95% CI upper**	***P***
Gender	0.756	0.461	1.240	0.268
Age	1.040	1.008	1.072	0.012
Education	0.974	0.716	1.323	0.864
Course of disease	1.007	0.974	1.042	0.680
Hba1c	1.063	0.915	1.235	0.424
Use of metformin	0.710	0.318	1.586	0.403
Metformin dosage	0.964	0.669	1.390	0.845
Nephropathy	0.704	0.288	0.717	0.440
Retinopathy	0.907	0.499	1.648	0.747
Coronary heart disease	1.375	0.729	2.595	0.326
Peripheral vascular disease	0.464	0.214	1.007	0.052
Dizziness, instability	0.977	0.534	1.788	0.941
Fullness	0.924	0.474	1.799	0.816
Hypertension	0.668	0.407	1.096	0.110
TG	0.978	0.831	1.152	0.793
				

**FIGURE 1:**
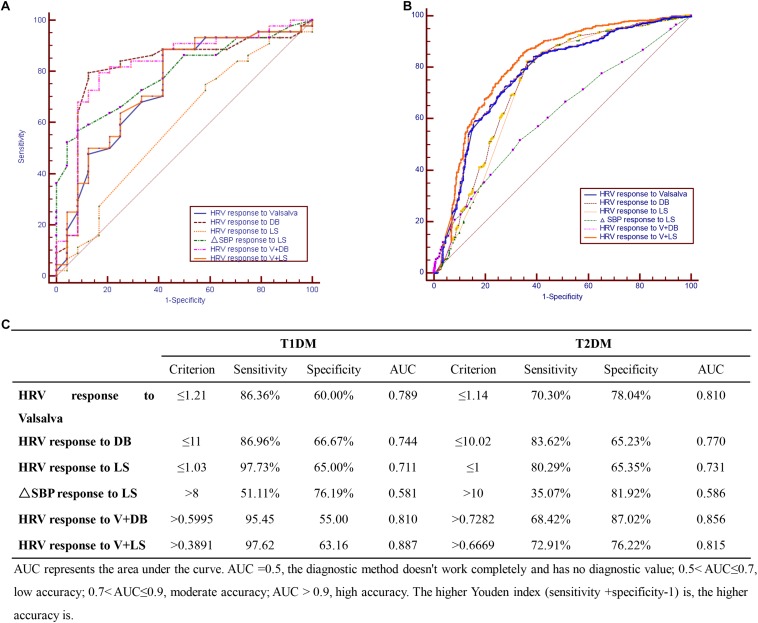
Cardiovascular autonomic neuropathy (CAN) diagnostic tests in **(A)** T1DM, **(B)** T2DM, and **(C)** combination of CAN diagnosis tests in T1DM and T2DM.

The four diagnostics tests were evaluated individually and in combination to find the optimal test (or test combinations) for CAN among the sampled Chinese diabetic patients. For the T1DM group, the optimal CAN diagnostic approach was combining the V and LS tests (sensitivity = 97.6%, AUC = 0.887) (Figures 1A,C); while for the T2DM group, the DB test had the highest sensitivity (83.6%) and a combination of V and DB tests gave the maximal AUC (0.856) (Figures 1B,C).

## Discussion

Many diabetic patients suffer from CAN without knowing it until, at times, the condition has progressed to late stage. Previous studies provide data about CAN prevalence in diabetic patients, mostly among Western populations (Zoppini et al., 2015). Few such studies have been conducted among the Chinese, hence the motivation for the present study.

The prevalence of CAN as found in this study is 63%. This observation corroborates what was reported by Chung et al. (2014), Tang et al. (2014), Zeng et al. (2014). Furthermore, risk factors associated with CAN were the duration of disease and age, respectively, in T1DM and T2DM participants. This observation corroborates that reported by other researchers (Tannus et al., 2014; Yun et al., 2018).

Patients taking beta-blocker drugs, which could have impacted on the outcomes of autonomic function tests, were excluded from the study. This explains the comparable low rate of coronary heart disease among CAN as opposed to non-CAN patients with T1DM. Similarly, the levels of TGs among patients with CAN was lower compared to those among the non-CAN patients.

Typical diagnosis of CAN relies on finding 2 or more abnormal results of autonomic function tests (Fidanci et al., 2015). We followed a similar approach and, to reduce bias, all tests were executed in the same room, by the same person and applying the same instruments and devices for the duration of the study.

Results from this study demonstrate that the occurrence of CAN among patients with diabetes is related to the duration of disease and the age of the patient. A correlation with the use of Metformin, HbA1c level, neuropathy, retinopathy, coronary heart disease, peripheral vascular disease, hypertension and TG level, although reported in previous studies (Witte et al., 2005), was not evident. This shows how complex and heterogeneous the cause and progression of CAN is.

Regression analysis showed that the HbA1c level was not an independent risk factor for both T1DM and T2DM patients. The HbA1c level was estimated during the first CAN screening and can only represent the blood glucose control over the past 3 months. Additionally, it is possible that glycemic variability may induce hypoglycemic stress leading to decreased HRV independent of glycemic control as estimated by HbA1c (Jaiswal et al., 2014).

Our study found that nearly 20% of T1DM patients with CAN also suffered from kidney disease. The connection between CAN and kidney disease in diabetic patients is rather complicated. Some reports demonstrate that CAN accelerates the progress of kidney disease in T1DM patients (Weinrauch et al., 1998; Burger et al., 2002). As reported also previously (Tahrani et al., 2014), we found kidney disease to be an independent risk factor for the prevalence of CAN in T2DM. In contrast, a large prospective observational study in which 388 T1DM cases were followed-up for 10 years did not find an association between CAN and kidney disease (Astrup et al., 2006). In supporting the relationship between CAN and kidney disease, the former has been shown to affect the glomerular filtration pressure, glomerular endothelial cell damage and erythropoietin secretion (Sundkvist and Lilja, 1993; Deicher and Horl, 2003; Iseki and Kohagura, 2007).

This study found that for T1DM, the optimal CAN diagnostic method is the use of the V test in combination with the LS test while for T2DM, the DB test had the highest sensitivity with combined V and DB tests giving the maximal AUC. Since the initial method described by Ewing et al. in distinguishing CAN from non-CAN among diabetic patients (Ewing et al., 1978), efforts aimed at simplifying CAN diagnosis have been explored. For example, Mustonen et al. (1989) showed that the Ewing tests could be simplified with the same test efficiency by reducing the number of tests to three: the Valsalva maneuver, deep breathing (DB), and isometric handgrip tests. Later, Stranieri et al. (2013) found out that the DB test was the optimal single test for CAN diagnosis, while adding some of the remaining tests could obtain additional accuracy.

From this study, we have therefore obtained insights into the prevalence of CAN and found the optimal methods for CAN diagnosis in the respective groups of diabetic patients. The study, however, is not without limitations. First, there is great discrepancy between the number of T1DM and T2DM patients, making direct comparisons of characteristics and observations between the two patient groups difficult. Moreover, we did not categorize the CAN cases according to the level of severity since our aim was to optimize the tests for CAN diagnosis and not to stage the condition. Besides, most of the patients included in our study were in their fifth and sixth decades of life, and hence, had long duration of diabetes. For this reason, the results obtained in this study may not be reflective of the general population of diabetic patients. Further studies involving a wider age spectrum, including younger patients, are required to further validate and verify our findings.

## Conclusion

The current study found the prevalence of diabetic patients suffering from CAN to be 63%. Further regression analysis demonstrated that the course of disease and age are independent risk factors of CAN in T1DM and T2DM, respectively. Moreover, a combination of V test and LS test was found to be optimal for CAN diagnosis in T1DM while in T2DM, combining the V and DB tests gave the best results. Larger prospective studies with longer follow-up periods are recommended to confirm these results.

## Author Contributions

QP, QL, WD, and DZ designed the study and performed the experiments. LQ, WH, LM, and HL analyzed and interpreted the data. YL and XL wrote the manuscript. AW and HY assisted with processing images. LG and XX performed the computational analysis and contributed to the preparation of the manuscript.

## Conflict of Interest

The authors declare that the research was conducted in the absence of any commercial or financial relationships that could be construed as a potential conflict of interest.

## References

[B1] Astrup A. S. Tarnow L. Rossing P. Hansen B. V. Hilsted J. Parving H. H. (2006). Cardiac autonomic neuropathy predicts cardiovascular morbidity and mortality in type 1 diabetic patients with diabetic nephropathy. *Diabetes Care* 29 334–339. 10.2337/diacare.29.02.06.dc05-1242 16443883

[B2] Burger A. J. D’elia J. A. Weinrauch L. A. Lerman I. Gaur A. (2002). Marked abnormalities in heart rate variability are associated with progressive deterioration of renal function in type I diabetic patients with overt nephropathy. *Int. J. Cardiol.* 86 281–287. 10.1016/s0167-5273(02)00346-7 12419567

[B3] Cha S. A. Yun J. S. Lim T. S. Min K. Song K. H. Yoo K. D. (2016). Diabetic cardiovascular autonomic neuropathy predicts recurrent cardiovascular diseases in patients with type 2 diabetes. *PLoS One* 11:e0164807. 10.1371/journal.pone.0164807 27741306 PMC5065186

[B4] Chen J. Yang S. B. Liu J. Tang Z. H. (2015). Diagnostic performance analysis for diabetic cardiovascular autonomic neuropathy based on short-term heart rate variability using Bayesian methods: preliminary analysis. *Diabetol. Metab. Syndr.* 7:74. 10.1186/s13098-015-0070-z 26366204 PMC4566203

[B5] Chung J. O. Cho D. H. Chung D. J. Chung M. Y. (2014). Physiological serum bilirubin concentrations are inversely associated with the prevalence of cardiovascular autonomic neuropathy in patients with type 2 diabetes. *Diabet. Med.* 31 185–191. 10.1111/dme.12338 24147832

[B6] Deicher R. Horl W. H. (2003). Anaemia as a risk factor for the progression of chronic kidney disease. *Curr. Opin. Nephrol. Hypertens.* 12 139–143. 10.1097/00041552-200303000-00003 12589173

[B7] DeLong E. R. DeLong D. M. Clarke-Pearson D. L. (1988). Comparing the areas under two or more correlated receiver operating characteristic curves: a nonparametric approach. *Biometrics* 44 837–845. 3203132

[B8] Didangelos T. Moralidis E. Karlafti E. Tziomalos K. Margaritidis C. Kontoninas Z. (2018). A comparative assessment of cardiovascular autonomic reflex testing and cardiac 123i-metaiodobenzylguanidine imaging in patients with type 1 diabetes mellitus without complications or cardiovascular risk factors. *Int. J. Endocrinol.* 2018: 5607208. 10.1155/2018/5607208 PMC5867537 29721015

[B9] Ewing D. J. Campbell I. W. Murray A. Neilson J. M. Clarke B. F. (1978). Immediate heart-rate response to standing: simple test for autonomic neuropathy in diabetes. *Br. Med. J.* 1 145–147. 10.1136/bmj.1.6106.145 620228 PMC1602825

[B10] Ewing D. J. Martyn C. N. Young R. J. Clarke B. F. (1985). The value of cardiovascular autonomic function tests: 10 years experience in diabetes. *Diabetes Care* 8 491–498. 10.2337/diacare.8.5.491 4053936

[B11] Fidanci M. K. Gulgun M. Genc A. (2015). Analysis of heart rate variability seems to be one step ahead of cardiac reflex tests for investigating cardiovascular autonomic neuropathy. *Anatol. J. Cardiol.* 15 849–850. 10.5152/anatoljcardiol.2015.6568 PMC5336976 26477724

[B12] Hazari M. A. Khan R. T. Reddy B. R. Hassan M. A. (2012). Cardiovascular autonomic dysfunction in type 2 diabetes mellitus and essential hypertension in a South Indian population. *Neurosciences* 17 173–175. 22465897

[B13] Iseki K. Kohagura K. (2007). Anemia as a risk factor for chronic kidney disease. *Kidney Int.* 72, (Suppl. 107), S4–S9. 17943141 10.1038/sj.ki.5002481

[B14] Jaiswal M. Mckeon K. Comment N. Henderson J. Swanson S. Plunkett C. (2014). Association between impaired cardiovascular autonomic function and hypoglycemia in patients with type 1 diabetes. *Diabetes Care* 37 2616–2621. 10.2337/dc14-0445 24973438 PMC4140160

[B15] Khoharo H. K. Halepoto A. W. (2012). QTc-interval, heart rate variability and postural hypotension as an indicator of cardiac autonomic neuropathy in type 2 diabetic patients. *J. Pak. Med. Assoc.* 62 328–331. 22755273

[B16] Maser R. E. Lenhard M. J. (2005). Cardiovascular autonomic neuropathy due to diabetes mellitus: clinical manifestations, consequences, and treatment. *J. Clin. Endocrinol. Metab.* 90 5896–5903. 10.1210/jc.2005-0754 16014401

[B17] May O. Arildsen H. (2000). Assessing cardiovascular autonomic neuropathy in diabetes mellitus: how many tests to use? *J. Diabetes Complicat.* 14 7–12. 10.1016/s1056-8727(00)00062-3 10925060

[B18] Mustonen J. Lansimies E. Uusitupa M. Talwar S. Hyodynmaa S. Karkkainen A. (1989). Testing of autonomic cardiovascular regulation–methodological considerations. *Clin. Physiol.* 9 249–257. 10.1111/j.1475-097x.1989.tb00977.x 2743743

[B19] Pafili K. Trypsianis G. Papazoglou D. Maltezos E. Papanas N. (2015). Simplified diagnosis of cardiovascular autonomic neuropathy in type 2 diabetes using Ewing’s battery. *Rev. Diabet. Stud.* 12 213–219. 10.1900/RDS.2015.12.213 26676669 PMC5397991

[B20] Philips J. C. Marchand M. Scheen A. J. (2011). Squatting, a posture test for studying cardiovascular autonomic neuropathy in diabetes. *Diabetes Metab.* 37 489–496. 10.1016/j.diabet.2011.09.004 22071282

[B21] Pop-Busui R. (2012). What do we know and we do not know about cardiovascular autonomic neuropathy in diabetes. *J. Cardiovasc. Transl. Res.* 5 463–478. 10.1007/s12265-012-9367-6 22644723 PMC3634565

[B22] Pop-Busui R. Braffett B. H. Zinman B. Martin C. White N. H. Herman W. H. (2017). Cardiovascular autonomic neuropathy and cardiovascular outcomes in the diabetes control and complications trial/epidemiology of diabetes interventions and complications (DCCT/EDIC) study. *Diabetes Care* 40 94–100. 10.2337/dc16-1397 27803120 PMC5180458

[B23] Stranieri A. Abawajy J. Kelarev A. Huda S. Chowdhury M. Jelinek H. F. (2013). An approach for Ewing test selection to support the clinical assessment of cardiac autonomic neuropathy. *Artif. Intell. Med.* 58 185–193. 10.1016/j.artmed.2013.04.007 23768975

[B24] Sundkvist G. Lilja B. (1993). Autonomic neuropathy predicts deterioration in glomerular filtration rate in patients with IDDM. *Diabetes Care* 16 773–779. 8495619 10.2337/diacare.16.5.773

[B25] Tahrani A. A. Dubb K. Raymond N. T. Begum S. Altaf Q. A. Sadiqi H. (2014). Cardiac autonomic neuropathy predicts renal function decline in patients with type 2 diabetes: a cohort study. *Diabetologia* 57 1249–1256. 10.1007/s00125-014-3211-2 24623102

[B26] Tang Z. H. Zeng F. Ye K. Yu X. Zhou L. (2014). The analysis of a reference value for baroreflex sensitivity and cardiovascular autonomic neuropathy prevalence in a chinese population. *Eur. J. Med. Res.* 19:8. 10.1186/2047-783X-19-8 24521230 PMC4004869

[B27] Tannus L. R. M. Drummond K. R. G. Clemente E. L. D. S. da Matta M. D. F. B. Gomes M. B. (2014). Predictors of cardiovascular autonomic neuropathy in patients with type 1 diabetes. *Front. Endocrinol.* 5:191. 10.3389/fendo.2014.00191 25505446 PMC4243695

[B28] Vinik A. I. Erbas T. Casellini C. M. (2013). Diabetic cardiac autonomic neuropathy, inflammation and cardiovascular disease. *J. Diabetes Investig.* 4 4–18. 10.1111/jdi.12042 PMC3580884 23550085

[B29] Vinik A. I. Ziegler D. (2007). Diabetic cardiovascular autonomic neuropathy. *Circulation* 115 387–397. 17242296 10.1161/CIRCULATIONAHA.106.634949

[B30] Voznesenskii B. B. (1965). [Combined functional tests as a method for studying the characteristics of autonomic regulation under conditions of changed body reactivity]. *Patol. Fiziol. Eksp. Ter.* 9 56–58. 5883747

[B31] Weinrauch L. A. Kennedy F. P. Gleason R. E. Keough J. D’elia J. A. (1998). Relationship between autonomic function and progression of renal disease in diabetic proteinuria: clinical correlations and implications for blood pressure control. *Am. J. Hypertens.* 11 302–308. 9544870 10.1016/s0895-7061(97)00472-x

[B32] Witte D. R. Tesfaye S. Chaturvedi N. Eaton S. E. Kempler P. Fuller J. H. (2005). Risk factors for cardiac autonomic neuropathy in type 1 diabetes mellitus. *Diabetologia* 48 164–171. 15619072 10.1007/s00125-004-1617-y

[B33] World Health Organization [WHO] (1999). *Report of a Who Consultation. Part 1: Diagnosis and Classification of Diabetes Mellitus.* Geneva: Definition and classification of diabetes mellitus and its complications.

[B34] Yun J.-S. Park Y.-M. Cha S.-A. Ahn Y.-B. Ko S.-H. (2018). Progression of cardiovascular autonomic neuropathy and cardiovascular disease in type 2 diabetes. *Cardiovasc. Diabetol.* 17:109. 10.1186/s12933-018-0752-6 PMC6071370 30071872

[B35] Zeng F. Tang Z. H. Li Z. Yu X. Zhou L. (2014). Normative reference of short-term heart rate variability and estimation of cardiovascular autonomic neuropathy prevalence in Chinese people. *J. Endocrinol. Invest.* 37 385–391. 10.1007/s40618-013-0047-4 24633734

[B36] Zoppini G. Cacciatori V. Raimondo D. Gemma M. Trombetta M. Dauriz M. (2015). Prevalence of cardiovascular autonomic neuropathy in a cohort of patients with newly diagnosed type 2 diabetes: the verona newly diagnosed type 2 diabetes study (VNDS). *Diabetes Care* 38 1487–1493. 10.2337/dc15-0081 26068862

